# Single versus double chest drains after pulmonary lobectomy: a systematic review and meta-analysis

**DOI:** 10.1186/s12957-020-01945-1

**Published:** 2020-07-20

**Authors:** Jinzhi You, Hailing Zhang, Wei Li, Ninghuang Dai, Zhongfeng Zheng

**Affiliations:** 1 Department of Cardiothoracic Surgery, The Affiliated Suqian Hospital of Xuzhou Medical University, No.138 South Huanghe Road, Sucheng District, Suqian, China; 2 The Suqian Clinical College of Xuzhou Medical University, Suqian, China

**Keywords:** Chest, Drain, Pulmonary lobectomy, Management, Treatment

## Abstract

**Background:**

Previous randomized controlled trials have compared the efficacy and safety of single chest drain (SCD) and double chest drains (DCD) in the patients undergone pulmonary lobectomy, yet the results remain inconsistent. Therefore, we aimed to conduct this present systematic review and meta-analysis to evaluate the role of SCD and DCD in the patients undergone pulmonary lobectomy.

**Methods:**

PubMed, Medline, EMBASE, Cochrane library, Web of Science, China National Knowledge Infrastructure, Wanfang, Weipu, and China Biomedical Literature databases were searched up to February 28, 2020, to identify the potential RCTs on SCD and DCD in the patients undergone pulmonary lobectomy. The main outcomes including verbal pain score, the duration of drainage (days), the length of hospital stay (days), and the incidence of air leak and re-drainage were collected and analyzed. All the data were processed and analyzed with software RevMan 5.3. We calculated and analyzed the odds ratios (OR) for dichotomous outcomes and the mean difference (MD) for continuous outcomes.

**Results:**

A total of 11 RCTs with 1214 patients were included, in which 589 patients received SCD treatment and 625 patients DCD treatment. The verbal pain score (MD = − 0.54, 95%CI (− 0.87, − 0.21)), the duration of drainage (MD = − 0.65, 95%CI (− 1.04, − 0.26)), and the length of hospital stay (MD = − 0.55, 95%CI (− 0.80, − 0.29)) in SCD group were significantly less than that of DCD group. There were no significant differences on the incidence of air leak (OR = 1.35, 95%CI (0.86, 2.11)) and re-drainage (OR = 0.88, 95%CI (0.41, 1.90)) among the two groups.

**Conclusions:**

SCD is a safe option, and it has the advantages of less postoperative pain, shortened duration of drain, and reduced length of hospital stay when compared with DCD in the patients undergone pulmonary lobectomy.

## Background

Lung cancer is one of the most common malignant tumors with high mortality [1]. In recent years, the morbidity and mortality have increased sharply year by year, which seriously harms human health [2]. Surgery is the main treatment for lung cancer, and closed chest tubes are placed generally for drainage. The purpose of placing closed chest drains after lobectomy is mainly to drain the blood and gas from the chest cavity and prevent regurgitation, to restore the normal negative pressure in the chest cavity, thereby promoting the lung expansion and prevent infections in the chest cavity [3]. The closed drainage can be used to observe whether there is active bleeding in postoperative patients, which is conducive to the recovery of postoperative pulmonary function and reduces the incidence of pulmonary infection [4, 5]. Therefore, the management of chest drains after pulmonary lobectomy is crucial to the prognosis of patients.

With the popularization of the concept of accelerated rehabilitation in recent years, more health care providers are endeavor to maximize the benefits of patients after surgery. There is currently controversy over the number of closed chest drainage tubes placed after surgery. The traditional chest drainage method after lobectomy is to place two drainage tubes. The main reason is that the double chest drains (DCD) can effectively drain the fluids, and it is safe and reliable to remove the tube either at the end of inspiration or at the end of expiration [6]. However, for single chest drain (SCD), it is necessary to quickly remove the tube after the end of inhalation to prevent the occurrence of pneumothorax [7]. It has also been reported that SCD can not only significantly reduce pain level after surgery but also can significantly reduce the drainages [8]. With the rise of the concept of rapid recovery, it is believed that DCD increases the pain and cost of patients, but whether the effect of SCD can achieve the effect of DCD remains to be further evaluated.

There are several randomized controlled trials (RCTs) [9–11] that have compared the role of SCD and DCD in the patients undergone pulmonary lobectomy, yet the results remain inconsistent. Several previous meta-analyses [8, 12–14] have focused on the effects and safety of SCD and DCD, but the sample size is rather small with lack of statistical testing power. Based on literature review, we have found that there are several recent related RCTs that have published. Therefore, it is necessary to conduct updated meta-analysis to identify the role of SCD and DCD in the patients undergone pulmonary lobectomy, thereby producing evidence for the management of pulmonary lobectomy.

The aim of this meta-analysis and systematic review was to evaluate the effects and safety of SCD and DCD in the patients undergone pulmonary lobectomy, to provide insights into the clinical management of pulmonary lobectomy.

## Methods

This present study was established and reported in accordance with the recommendations and guidance of Preferred Reporting Items for Systematic Reviews and Meta-Analyses (PRISMA) [15].

### Literature research

The literature researches were conducted in comply with the recommendations [16] of literature search in surgical systematic reviews. Two authors independently searched PubMed, Medline, EMBASE, Cochrane library, Web of Science, China National Knowledge Infrastructure (CNKI), Wanfang, Weipu, and China Biomedical Literature databases. The time range was set from the chest tube insertion to February 28, 2020. All RCTs comparing SCD and DCD in the patients undergone pulmonary lobectomy were identified and included. The search strategy was built as (“lobectomy” OR “chest” OR “thoracic”) AND (“drain” OR “tube” OR “drainage” OR “single” OR “one” OR “double” OR “two”) in every database. All relevant titles and abstracts were imported to the Endnote software for selection. In addition to the use of electronic searches, some conference papers and references were manually searched. Two authors conducted systematic evaluation of all identified articles based on inclusion and exclusion criteria.

### The inclusion and exclusion criteria

The studies were included if they met every item of the following criteria: (1) RCT design; (2) patient underwent lobectomy without radiotherapy or chemotherapy before surgery, and it was the first time for the surgery in thoracic systems; (3) the study has compared the SCD and DCD; and (4) related outcomes such as the pain level after surgery, the duration of drains, etc. had reported, and the data can be extracted for data analysis. The studies were excluded if: (1) retrospective analysis, case report, reviews, etc. non-RCT design studies; (2) duplicate reports; and (3) patients undergone other surgeries concurrently.

### Data extraction

Data extraction was performed independently by two authors. We developed a unified form to collect relevant data. We extracted the following information: first author, year of publication, type of study, the population of included participants, gender, age, type of surgery, and related reported outcomes including verbal pain score, the duration of drainage (days), the length of hospital stay (days), and the incidence of air leak and re-drainage. We discussed with the third author if there is a disagreement during the data extraction process.

### Risk of bias assessment

The assessments of the quality of included RCTs were conducted with the bias risk assessment tools recommended by the Cochrane Collaboration [17]. The main contents included sequence generation, allocation concealment, blinding of participants and personnel, blinding of outcome assessment, incomplete outcome data, selective outcome reporting, and “other” issues. Each domain was defined as low, middle, and high risk of bias according to the criteria. The presence or absence of blinding of the study contributors—patients, surgeons, data collectors, outcome assessors, and data analysts—was analyzed as previously suggested [18].

### Statistical analysis

All the data were processed and analyzed with the software RevMan 5.3 recommended by the Cochrane library. We calculated and analyzed the odds ratios (OR) for dichotomous outcomes and the mean difference (MD) for continuous outcomes. The level of heterogeneity among the analyzed RCTs was evaluated with the *I*^2^ statistics (*I*^2^ > 50% indicating significant heterogeneity). And the fixed-effects model was applied if there was no statistically significant heterogeneity (*p* ≥ 0.10, *I*^2^ < 50%); otherwise, random-effects model was used (*p* < 0.10, *I*^2^ > 50%). Sensitivity analysis was performed by removing one by one to see the changes of whole synthesized results. Besides, funnel plots and Begg’s tests were applied to assess the publication bias. *p* < 0.05 was considered as being statistically significant. For all the statistical analyses in this present meta-analysis, *p* < 0.05 was considered as statistical significance, and all the detected tests were two-sided.

## Results

### Study inclusion

**Fig. 1:**
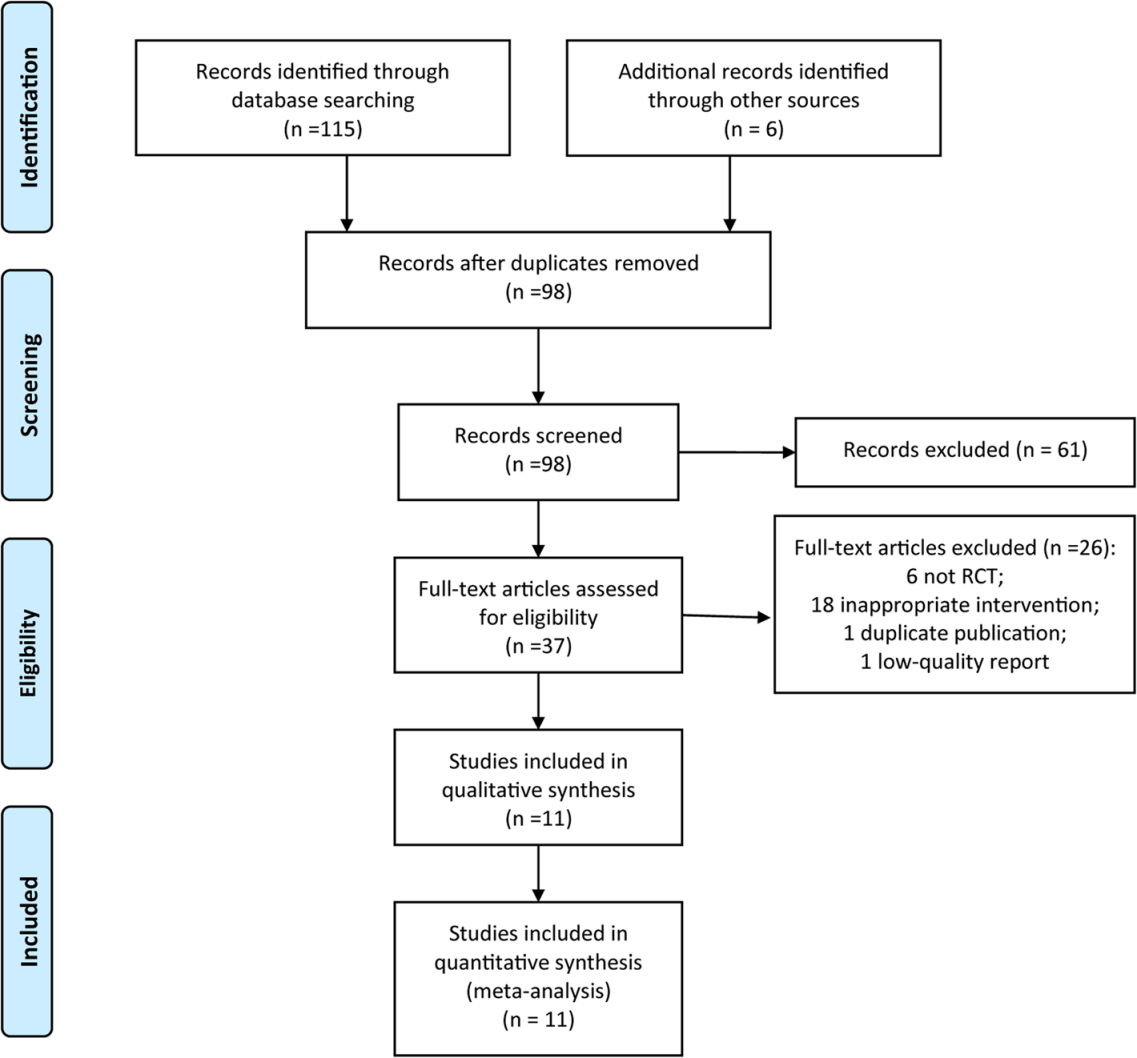
The flow chart of study selection

The process of study inclusion is presented in Fig. 1. The first search resulted in 121 potentially relevant articles. After reviewing the titles and abstracts of the remaining 98 studies after duplicate exclusion, the full texts of 37 studies were retrieved. After further evaluation, 11 RCTs [9–11, 19–26] were included finally.

### The characteristic of included RCTs

A total of 11 RCTs [9–11, 19–26] with 1214 patients with pulmonary lobectomy were included, specifically 589 patients undergoing SCD treatment and 625 patients undergoing DCD treatment. The characteristics of included 11 RCTs were presented in Table 1. The six included RCTs [11, 19, 20, 22, 25, 26] were conducted in China, one in the UK [9], Spain [10], Turkey [21], Poland [23], and Japan [24], respectively. The sample size ranged from 183 to 43. And the pulmonary lobectomy varied from different part of lung tissue.

**Table 1:** The characteristics of included RCTs

Study	Country	Sample size		Gender (male/female)		Age		Follow-up period	The type of surgery
		SCD	DCD	SCD	DCD	SCD	DCD		
Alex et al. [9]	UK	60	60	48/12	48/12	65 ± 8.4	66 ± 8.6	Those patients with chest drains were assessed every hour for the first 24 h and then every 6 h until removal.	Lobectomy
Gomez-Caro et al. [10]	Spain	60	59	9/51	7/52	65.5 ± 9.4	61.5 ± 9.5	30 days	Upper and lower lobe resection
Han et al. [11]	China	46	47	39/7	32/15	58.4 ± 9.5	58.2 ± 9.0	Not available	Lobectomy
Hu et al. [19]	China	62	53	32/30	27/26	61.3	60.1	Not available	Left and right upper lobe resection plus lymph node dissection
Li et al. [20]	China	21	22	12/9	13/9	56.5 ± 9.5	57.2 ± 9.0	Until discharge	Upper lobe resection
Okur et al. [21]	Turkey	50	50	37/13	43/7	54.74 ± 14.34	56.34 ± 11.52	Until discharge	Lobectomy
Pan et al. [22]	China	48	48	26/22	28/20	56.37 ± 6.28	52.18 ± 5.63	30 days	Upper lobe resection
Pawelczyk et al. [23]	Poland	90	93	64/26	54/39	60.9 ± 9.03	60.7 ± 8.906	Not available	Upper and lower lobe resection
Tanaka et al. [24]	Japan	54	54	38/16	32/22	66.8 ± 7.5	67.7 ± 8.0	Not available	Upper and lower lobe resection
Wang et al. [25]	China	38	79	19/19	45/34	63.98 ± 9.76	65.77 ± 8.58	Until discharge	Upper lobe resection
Zhou and Yu [26]	China	60	60	41/19	39/21	50.83 ± 15.23	52.55 ± 13.23	Until discharge	Lobectomy

### The quality of included RCTs

**Fig. 2:**
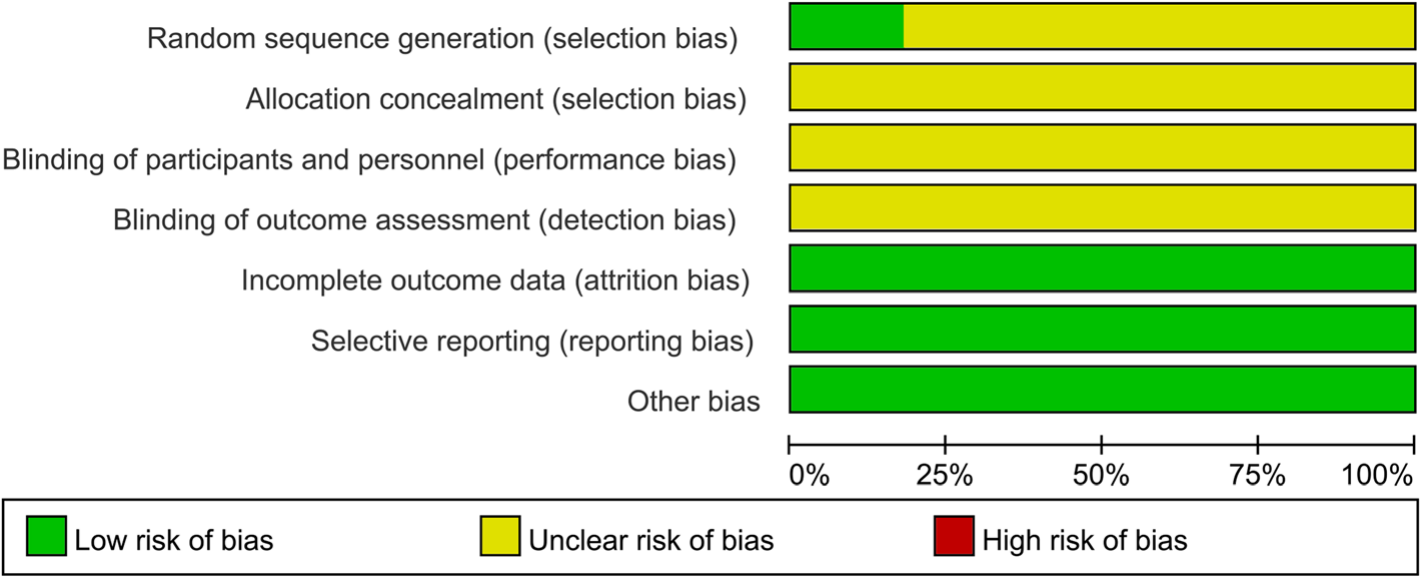
Risk of bias graph

**Fig. 3:**
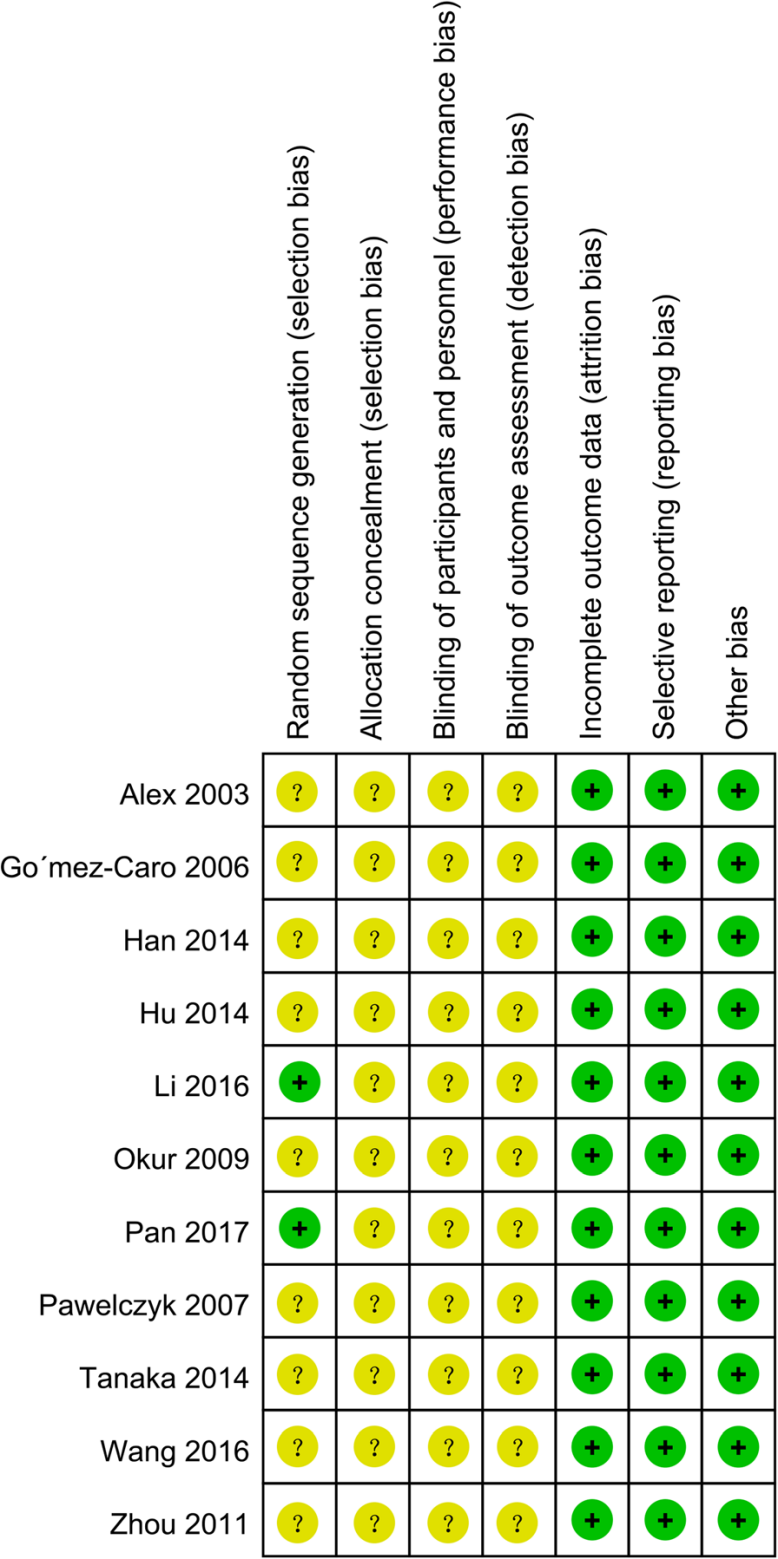
Risk of bias summary

The quality of included RCTs was showed in Figs. 2 and 3. Although every included study mentioned randomization, only three studies [20, 22, 24] have reported the methods used for random number generation. One RCT [24] reported the blinded information regarding the group assignment and thoracentesis operation. None of the resting ten RCTs explicitly reported the distribution and concealment or any blind design among the method part. And no other significant biases were found.

### The meta-analysis of extracted outcomes

#### The verbal pain score

**Fig. 4:**
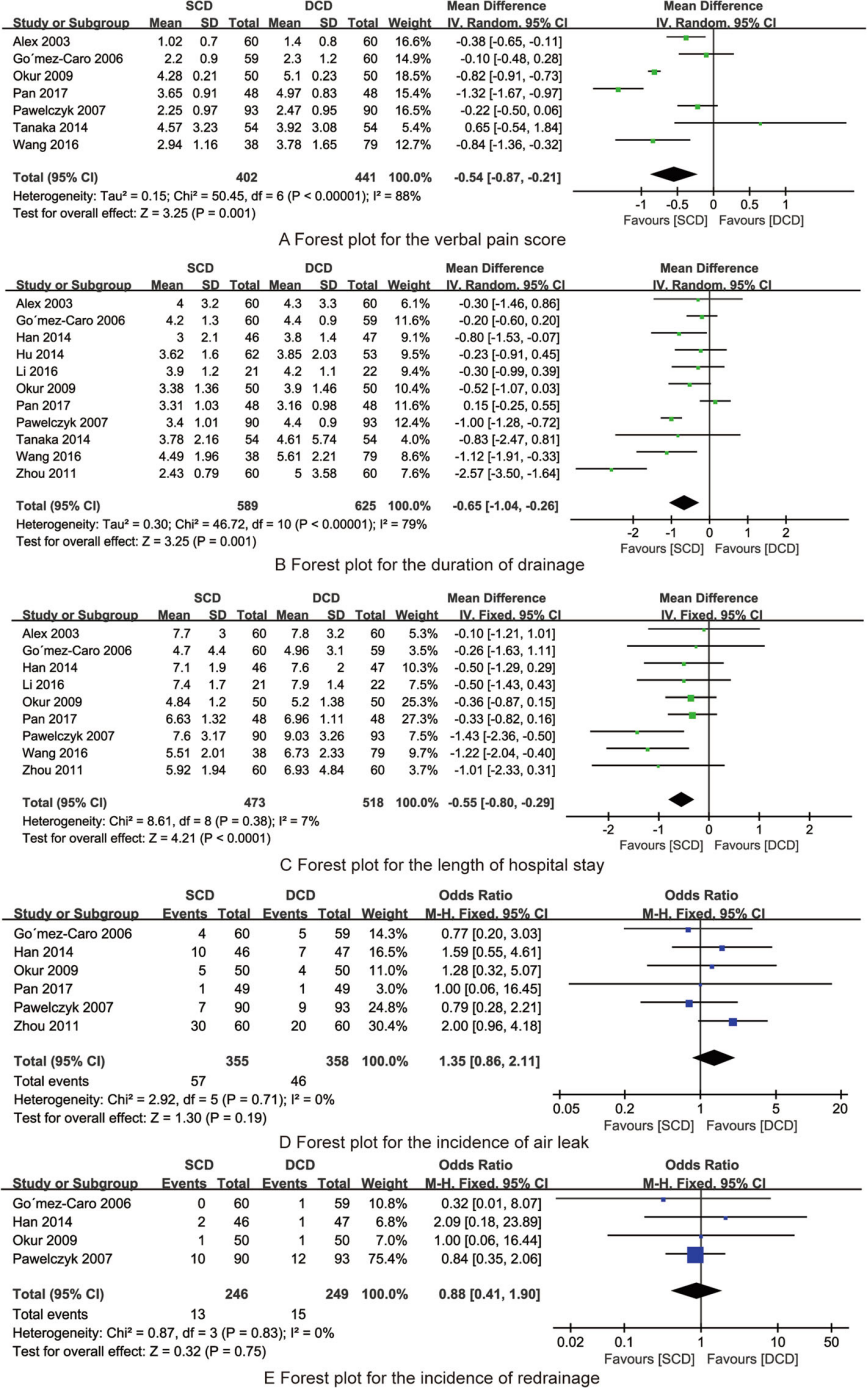
The forest plot for the synthesized outcomes

Seven included RCTs [9, 10, 21–25] reported the verbal pain score after pulmonary lobectomy. Significant heterogeneity was found among included RCTs (*I*^2^ = 88%), and random model was applied. The analysis result showed that the verbal pain score in SCD group was significantly less than that of DCD treatment (MD = − 0.54, 95%CI (− 0.87, − 0.21), Fig. 4a).

#### The duration of drainage (days)

All the included RCTs [9–11, 19–26] reported the duration of drainage (days) after pulmonary lobectomy. Significant heterogeneity was found among included RCTs (*I*^2^ = 79%), and random model was applied. The analysis result showed that the duration of drainage (days) in SCD group was significantly less than that of DCD treatment (MD = − 0.65, 95%CI (− 1.04, − 0.26), Fig. 4b).

#### The length of hospital stay (days)

Nine included RCTs [9–11, 20–23, 25, 26] reported the length of hospital stay (days) after pulmonary lobectomy. No significant heterogeneity was found among included RCTs (*I*^2^ = 7%), and fix model was applied. The analysis result showed that length of hospital stay (days) in SCD group was significantly less than that of DCD treatment (MD = − 0.55, 95%CI (− 0.80, − 0.29), Fig. 4c).

#### The incidence of air leak

Six included RCTs [10, 11, 21–23, 26] reported the incidence of air leak after pulmonary lobectomy. No significant heterogeneity was found among included RCTs (*I*^2^ = 0%), and fix model was applied. The analysis result showed that there was no significant difference on the incidence of air leak in SCD and DCD group (OR = 1.35, 95%CI (0.86, 2.11), Fig. 4d).

#### The incidence of re-drainage

Four included RCTs [10, 11, 21, 23] reported the incidence of re-drainage after pulmonary lobectomy. No significant heterogeneity was found among included RCTs (*I*^2^ = 0%), and fix model was applied. The analysis result showed that there was no significant difference on the incidence of re-drainage in SCD and DCD group (OR = 0.88, 95%CI (0.41, 1.90), Fig. 4e).

### Sensitivity analysis

The sensitivity analyses were conducted by excluding single RCT one by one and excluded the studies from the same countries; the results of sensitivity analysis of all outcomes had showed no significant result changes among the overall estimates.

### Publication bias

**Fig. 5:**
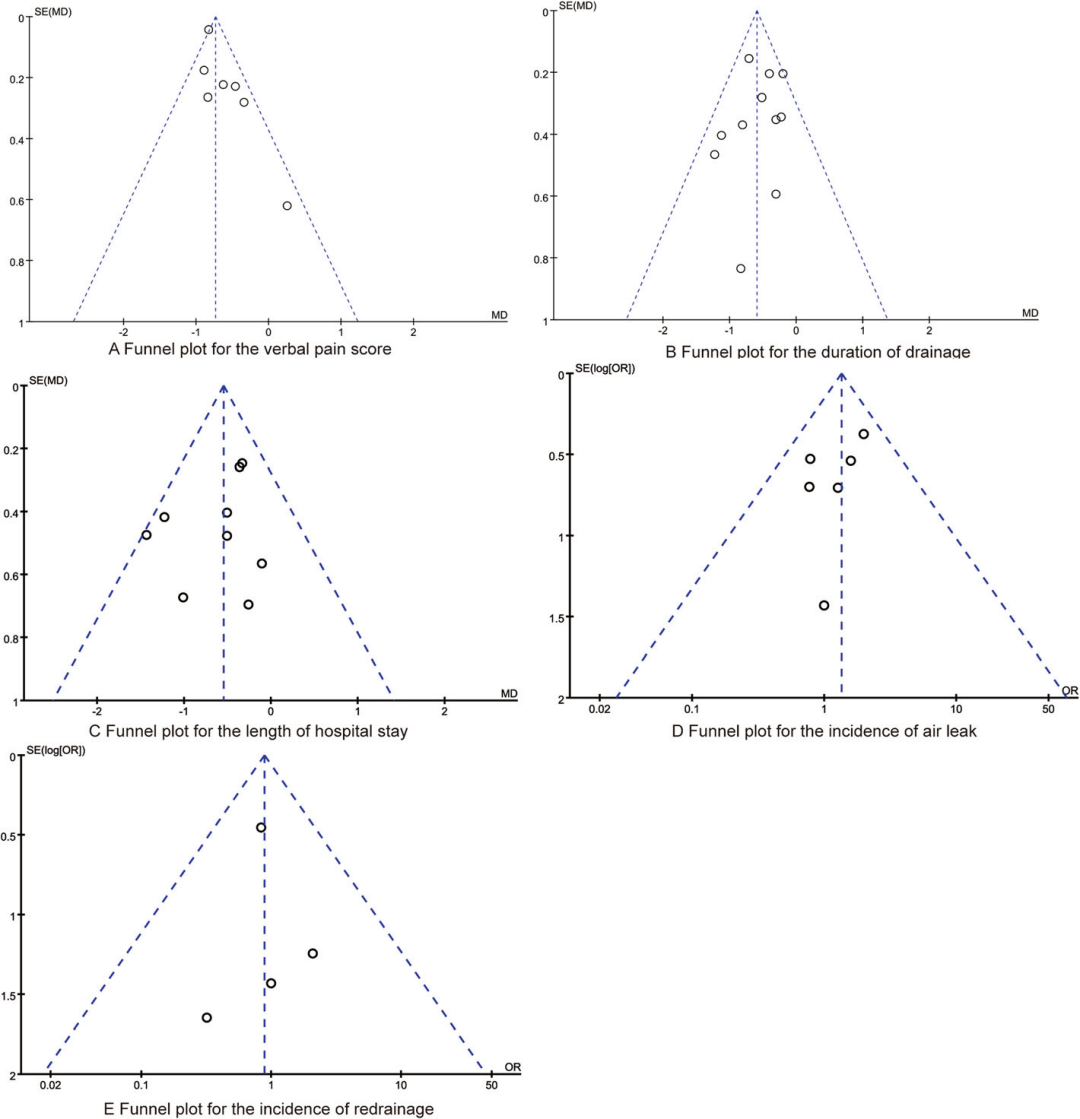
The funnel plots for synthesized outcomes

The publication bias was assessed with funnel plot in this present study. The funnel plots (Fig. 5) for all synthesized outcomes remained symmetrical, indicating no visual signs of publication bias among the included studies.

## Discussion

The application of thoracic drainage after lobectomy has been in the clinic for many years. By placing a closed chest drainage tube, it can achieve the effects of draining gas and fluids from the chest cavity, promoting lung expansion, preventing chest infections, and rebuilding negative pressure [27, 28]. Meanwhile, by observing the amount, characteristics of drainages, and the fluctuation of the water column, some abnormal conditions can be found and treated in time [29]. At present, it is common that SCD is placed after lobectomy of upper lung tissue in China, while DCD are placed after lobectomy of lower lung tissue [14]. The potential reason is that the closure of the residual cavity after lobectomy depends on adequate drainage with aims to promote lung expansion and avoid mediastinum displacement [30]. The length of the drain inserted into the chest cavity is easily restricted [31]. If it is placed too high, the pleural fluid located below it cannot be easily drained. However, placing it too low can be folded up by the pressure of the diaphragm to bend and affect the drainage output [32]. Sometimes it can also stimulate the diaphragm to cause stubborn hiccups or severe pain during breathing, resulting in limited breathing [33]. We have conducted a meta-analysis of included RCTs comparing the effects and safety of SCD and DCD after pulmonary lobectomy, and we have found that the pain level in SCD is much less than that of DCD, and the duration of drainage and hospital stay are much shorter than DCD, and SCD and DCD do not act different role in the incidence of air leak and re-drainage. Therefore, SCD may be more advantageous in the management of patients undergone pulmonary lobectomy.

There are also reports that SCD can not only significantly reduce the pain but also can significantly reduce drainage, which is beneficial for patients to go to the ground early and exercise early to promote the recovery of respiratory function [34, 35]. Previous studies [13, 36, 37] have indicated that the effect of SCD is better than or equivalent to traditional DCD drainage, and it does not increase the incidence of complications and mortality. Furthermore, the clinical advantages of SCD also include that it is conducive to skin incision healing [38]. It is noteworthy that SCD is generally placed at the top of the chest, even though related studies [39, 40] have shown that the main advantages of SCD after lobectomy have nothing to do with surgical approaches; there are few reports on SCD placement in the epilobectomy; future studies on this type of surgery are needed.

The drainage volume of the SCD is less than that of DCD; there are few possible reasons for the decrease of drainages in SCD. Firstly, the duration of the SCD is much shorter than that of DCD; the increased pleural secretion in DCD can be explained by more pleural fluid secretion from the pleura with regard to more irritation of DCD [41]. Secondly, more advanced instruments and materials are used in lung resection nowadays, such as linear staplers for incomplete fissures, sealing materials for substantial air leaks, or other advanced materials for hemostasis. All of those advancements promote the use of SCD and ensure the effects and safety of SCD.

This present meta-analysis has certain limitations that should be considered. Firstly, the quality of the included literature varies, and the analysis found that several outcomes have significant heterogeneity; the potential source of bias cannot be analyzed due to limited data. Secondly, the type of the drains and the methods of the pulmonary lobectomy among the included RCTs are different; the meta-analysis result may be influenced. Thirdly, our review is limited by the methodological quality of included RCTs; some information related to quality assessment is insufficient to make defined and solid judgment. Blinding reduces performance and detection bias in RCTs. There is evidence that lack of blinding leads to overestimation of treatment effects in trials [18]. Since surgical trials use interventions with a physical component, blinding is often complicated. Therefore, future studies with rigorous design and appropriate reporting format are warranted.

## Conclusions

In conclusion, the results of this present meta-analysis have found that SCD is much better than DCD in reducing the postoperative pain level, shortening the duration of drains, and decreasing the length of hospital stay. At the moment, there is just no convincing evidence that more than one drainage has any benefit. Therefore, SCD is a better option for patients undergone pulmonary lobectomy. However, we have not found any differences in the related complications such as the incidence of air leak and re-drainage between SCD and DCD, which may be associated with insufficient sample size. Therefore, large-sample and multicenter RCTs on the role of SCD and DCD are highlighted in the future.

## Data Availability

All data generated or analyzed during this study are included in this published article.

## Abbreviations

SCD: Single chest drain
DCD: Double chest drains
RCTs: Randomized controlled trials
PRISMA: Preferred Reporting Items for Systematic Reviews and Meta-Analyses
CNKI: China National Knowledge Infrastructure
OR: Odds ratios
MD: Mean difference
